# MiMiCPy: An Efficient Toolkit for MiMiC-Based QM/MM Simulations

**DOI:** 10.1021/acs.jcim.2c01620

**Published:** 2023-02-22

**Authors:** Bharath Raghavan, Florian K. Schackert, Andrea Levy, Sophia K. Johnson, Emiliano Ippoliti, Davide Mandelli, Jógvan Magnus Haugaard Olsen, Ursula Rothlisberger, Paolo Carloni

**Affiliations:** † Computational Biomedicine, Institute of Advanced Simulations IAS-5/Institute for Neuroscience and Medicine INM-9, Forschungszentrum Jülich GmbH, Jülich 52428, Germany; ‡ Department of Physics, RWTH Aachen University, Aachen 52074, Germany; ¶ Laboratory of Computational Chemistry and Biochemistry, Institute of Chemical Sciences and Engineering, École Polytechnique Fédérale de Lausanne (EPFL), CH-1015 Lausanne, Switzerland; § DTU Chemistry, Technical University of Denmark, DK-2800 Kongens Lyngby, Denmark

## Abstract

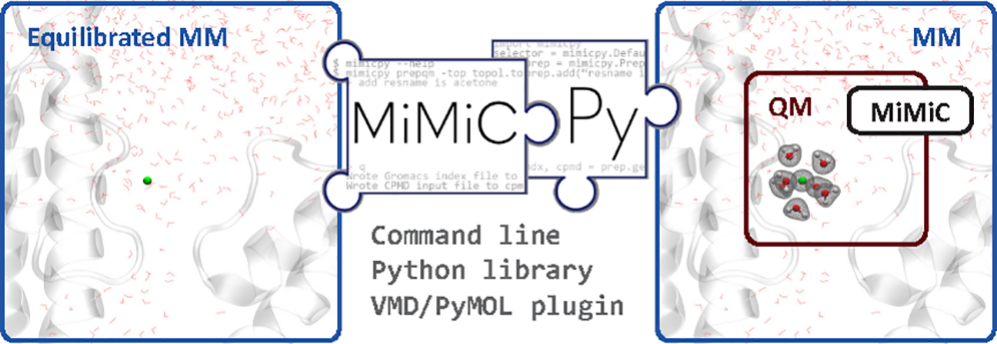

MiMiC is a highly flexible, extremely scalable multiscale modeling framework. It couples the CPMD (quantum mechanics, QM) and GROMACS (molecular mechanics, MM) codes. The code requires preparing separate input files for the two programs with a selection of the QM region. This can be a tedious procedure prone to human error, especially when dealing with large QM regions. Here, we present MiMiCPy, a user-friendly tool that automatizes the preparation of MiMiC input files. It is written in Python 3 with an object-oriented approach. The main subcommand PrepQM can be used to generate MiMiC inputs directly from the command line or through a PyMOL/VMD plugin for visually selecting the QM region. Many other subcommands are also provided for debugging and fixing MiMiC input files. MiMiCPy is designed with a modular structure that allows seamless extensions to new program formats depending on the requirements of MiMiC.

## Introduction

Biochemical processes span a wide range of time and length scales and often require explicit modeling of electronic degrees of freedom.^1^ These include enzymatic reactions, photobiological processes, and transition-metal ion binding to biomolecules.^2−4^ Currently, the most accurate and computationally expedient way to describe these processes is provided by hybrid quantum mechanics/molecular mechanics (QM/MM) multiscale approaches.^5−7^ Here, the system is split into a relatively small QM subsystem and a larger MM subsystem, which are treated at different levels of theory either by different programs (loose-coupling scheme) or within the same program (tight-coupling scheme).^8^ These methods offer an excellent trade-off between accuracy and computational cost. However, the accessible time scales in QM/MM molecular dynamics (MD) simulation are currently limited, especially when applying first-principles methods like density functional theory,^9^ which in turn affect the sampling accuracy of QM/MM MD.^10^

To alleviate this problem, the MiMiC^11^ framework for multiscale modeling in computational chemistry has been developed. MiMiC is based on a loose-coupling scheme without compromising computational efficiency. This flexibility allows for a relatively straightforward incorporation of any QM and MM code. The current release of MiMiC^12,13^ connects CPMD^14^ with GROMACS^15,16^ enabling massively parallel QM/MM MD simulations.^17^ In addition, support for CFOUR will be available soon, allowing for high-level wave function-based QM/MM simulations.^18^ MiMiC has displayed excellent scalability over more than thousands of cores, paving the way toward routine subnanosecond QM/MM MD of large biological systems.^19,20^

MiMiC QM/MM requires one input file for GROMACS and one for CPMD. The definition of the QM region must be added to both. To automatize this lengthy and error-prone task, we have developed MiMiCPy, a suite of tools for the smooth preparation of input files. MiMiCPy is based on Python 3^21^ and uses NumPy^22^ and Pandas^23^ for efficient data manipulation. It selects complex QM regions through an intuitive language, automatically tracking the atom index conversion between GROMACS and CPMD. MiMiCPy is designed with a modular approach, allowing it to be extended to handle the topology, coordinates, and input script formats of new MM and QM programs supported by MiMiC in the future.

This article is organized as follows. First, we present the procedure to prepare MiMiC input files. Next, we detail the usage of MiMiCPy. Finally, we provide practical examples for (bio)chemical systems.

## Implementation

Input files can be prepared in three different ways:

1. A set of command-line subcommands, including PrepQM and others that will be detailed below; this is the most convenient way to use MiMiCPy.
2. The PrepQM plugins for the VMD^24^ and PyMOL^25^ packages; this is ideal for selecting complex QM regions where the default selection language of MiMiCPy may not be so convenient or where visual inspection is required.
3. The Python library, which can be imported directly in a Python script, exposing all the features of MiMiCPy; this approach is powerful when developing automated workflows.

## Command Line and Plugins

**Figure 1:**
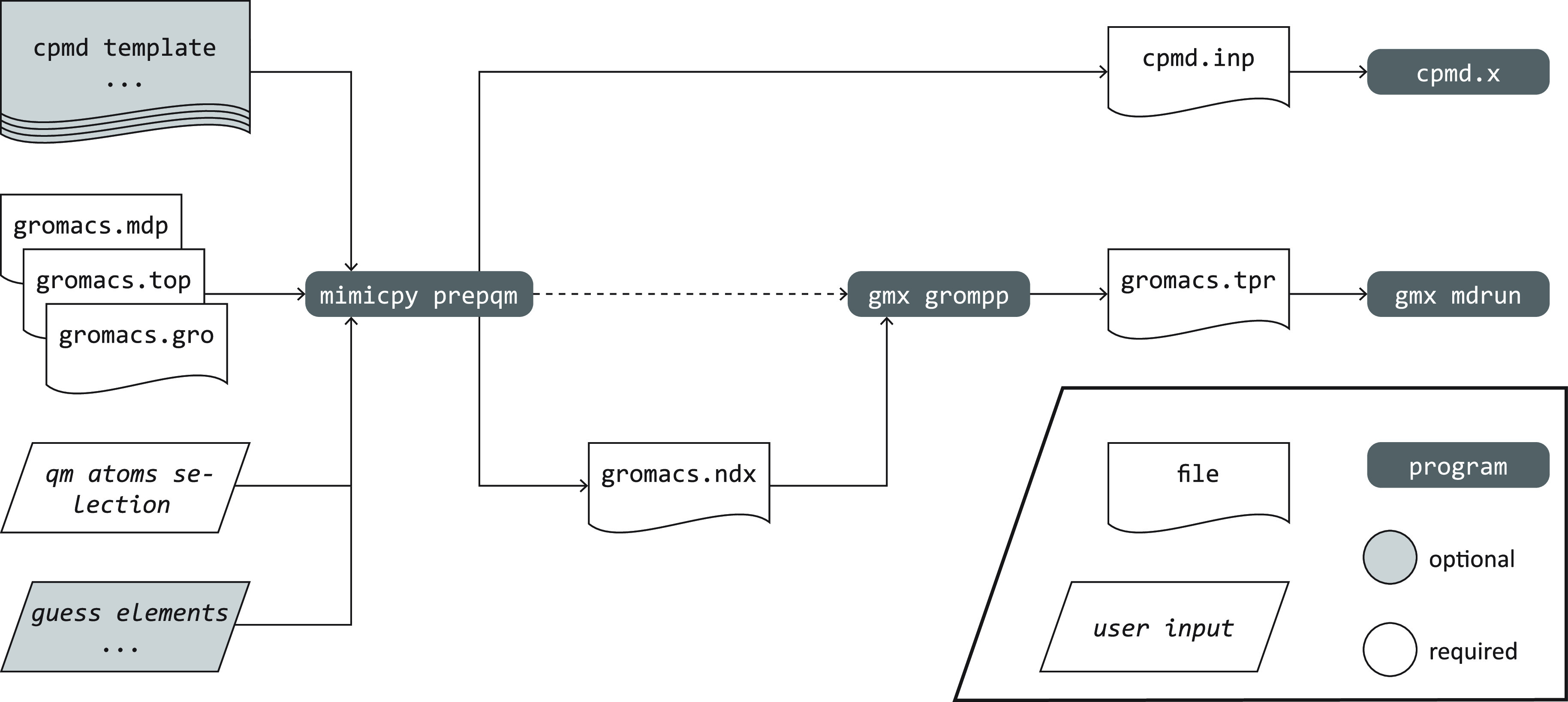
Flowchart of the generation of the CPMD and GROMACS input files for a MiMiC-based QM/MM simulation.

PrepQM is the chief subcommand used to generate both the GROMACS portable binary run (tpr) input file and the CPMD input file for a MiMiC QM/MM run. A workflow diagram depicting the general scheme of how input files are generated with MiMiCPy is shown in Figure 1.

The minimal set of arguments to be passed to the PrepQM subcommand are the topology and coordinate files. Currently, MiMiCPy supports GROMACS topology (.top), GROMACS gro (.gro), pdb (.pdb), xyz (.xyz), and the CPMD GEOMETRY format. Launching the mimicpy prepqm command starts an interactive session, where the atoms to be included in the QM region can be selected. This is done by using a custom selection language provided by MiMiCPy, which is designed to be human readable similar to the ones offered by CHARMM,^26^ VMD,^24^ and PyMOL.^25^ It includes selections by atom/residue properties grouped by Boolean operators. The syntax for the selection query involves the following general structure:
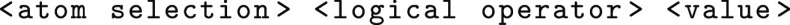
where atom selection can include resname for the residue name, resid for residue ID, name for the atom name, type for the atom type, id for the atom ID, and mol for the molecule/chain. All the IDs and names are as per conventions of the MM engine, i.e., the GROMACS topology. Logical operators can be is, not, >, <, ≥, or ≤. Many selection queries can be strung together using the and or or operators and grouped with brackets. In the interactive session, atoms can be added and/or deleted to the QM region. Examples of this are discussed in the Applications section.

PrepQM generates a CPMD input file with a minimum box size and the total charge. The &MIMIC and &ATOMS sections are also filled up. Other CPMD instructions can also be added.

A GROMACS index file, containing the GROMACS indices of the QM atoms, is also written by PrepQM. This file, with the topology, the coordinate file, and the molecular dynamics parameters (mdp) file, can be passed to the GROMACS preprocessor (gmx grompp) to generate the GROMACS tpr file. The same coordinate and topology files passed to PrepQM must be passed to gmx grompp. Conveniently, if a GROMACS mdp file is initially passed to PrepQM, it will call gmx grompp and generate the tpr file automatically. The generated CPMD input and the GROMACS tpr files are used to run the MiMiC-based QM/MM simulation by passing them to cpmd.x and gmx mdrun, respectively.

Selecting QM atoms through the command line may be inconvenient, especially for large QM regions. MiMiCPy provides PrepQM plugins for the VMD and PyMOL packages to select the QM region visually. Furthermore, the MiMiCPy console application provides other tools to fix and debug input files:

- CPMD2Coords writes the QM atoms selected in a MiMiC-compliant CPMD input file to a gro or pdb file.
- FixTop fixes missing information in GROMACS topology files that are required by CPMD in a MiMiC run.
- CPMDid provides the indices that CPMD assigns to each atom; this is especially useful for the MM atoms because in general such indices are reshuffled in a nonobvious way with respect to the GROMACS ordering.
- Geom2Coords converts a CPMD GEOMETRY file to a gro or pdb file for easy visualization.

## MiMiCPy as a Python Library

**Figure 2:**
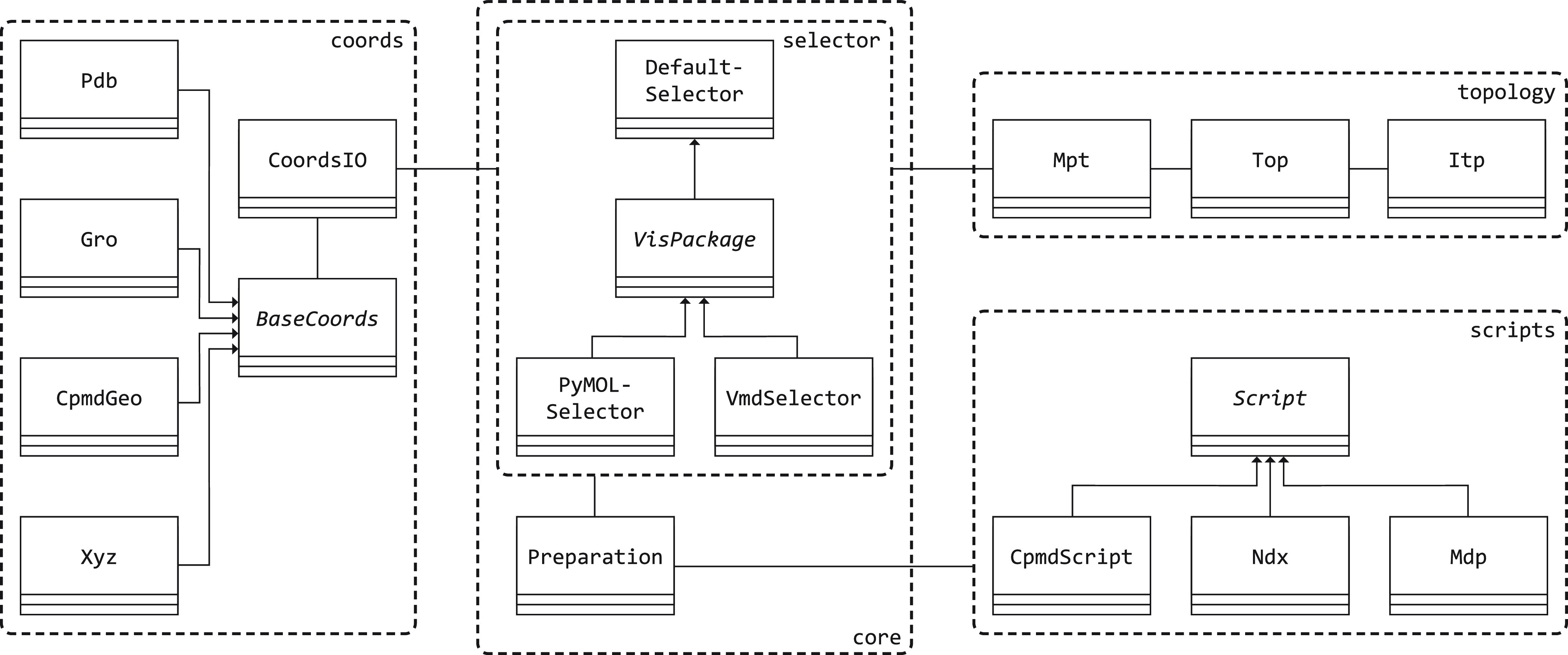
Organization of the main classes in MiMiCPy.

MiMiCPy can also be used as a Python library (Figure 2).

Coordinate and topology data can be loaded into MiMiCPy by using the dedicated CoordsIO and Mpt or MiMiCPy topology classes. These handle different coordinate and topology formats by passing the information to dedicated parser classes. Each coordinate file format has its own dedicated class which is inherited from BaseCoords. This is an abstract base class, providing the skeleton of a coordinate parser. Currently, MiMiCPy includes Gro, Pdb, CPMDGeo, and Xyz classes to handle the respective formats. The CoordsIO class acts as an adapter that aggregates and wraps these different classes and exposes only the coordinate information (as a Pandas DataFrame) and the box size to the user. This is the only information required by the rest of the package. The Mpt class functions in a similar way, as an adapter interfacing multiple topology parser classes. The Mpt class provides a common framework to deal with disparate topology formats. Mainly, it exposes methods for selecting specific atoms from the topology. Currently, only the GROMACS topology format (.top) is supported. Other formats may easily be supported by adding new classes that interface with the Mpt class.

The coordinate and topology information should be passed to a “Selector”-type class. This type of class combines coordinate and topology information, handling the selection of the atoms. To use the MiMiCPy selection language, an object of the DefaultSelector class can be instantiated. To use the selection languages of VMD and/or PyMOL, instances of the VmdSelector or PyMOLSelector classes can be created instead. These latter classes provide a simplified façade to the VMD and PyMOL software packages in order to work with the Preparation class (described below). Moreover, these classes are inherited from the abstract VisPackage class (which in turn is inherited from DefaultSelector), which provides a common set of rules for all these façade classes. A new façade class can be easily added to allow MiMiCPy to interface with other molecular visualization packages.

An instance of the desired selector-type class is to be passed to the Preparation class, which is the “central” class that keeps track of all the selected QM atoms and creates the input files. The Preparation class can be essentially thought of as a decorator for the selector classes, aggregating them and attaching the new behavior of input file generation. Calling the get_mimic_input() method of a Preparation instance returns instances of type CpmdScript and Ndx. These are children of the abstract Script class, allowing for “pythonic” interactions with these script instances, i.e., using the dot operator for setting and getting of script properties. Similarly, get_mimic_input() would result in a template Mdp object (GROMACS .mdp handler), which also is inherited from the Script class. All Script instances can be converted to and from text files.

MiMiCPy is fully object-oriented and built with a modular architecture in mind. It can be seamlessly extended to support new coordinate and topology formats. This allows it to quickly keep up with and support new developments in the MiMiC framework.

## Usage

The simplest way to create input files uses PrepQM:
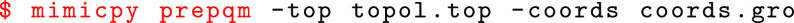
This command passes the GROMACS topology file topol.top and the initial coordinate file coords.gro to the PrepQM subprogram. The command starts an interactive session, where instructions can be given to add and/or delete atoms to select the QM region. An example of such an instruction is
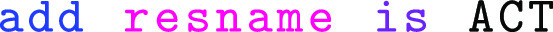
The keyword after add corresponds to the query that identifies the atoms to be added to the QM region. In this case, atoms in the residue with name ACT are selected. After selecting the desired atoms, typing q will exit the interactive session. MiMiCPy generates the CPMD input file cpmd.inp and the GROMACS index file index.ndx. The latter is used to generate the GROMACS tpr file. If the GROMACS mdp file is passed to PrepQM, this is done automatically:
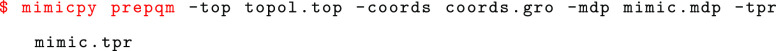
Other options are available in the PrepQM subcommand to tailor the input files to the user’s needs (see https://mimic-project.org/).

The species of the QM atoms need to be passed from GROMACS to CPMD. For standard atom types (e.g., atoms in the natural amino acids), the information is in the GROMACS force field. For nonstandard atom types (e.g., a ligand), this information is usually not found. MiMiCPy automatically guesses the atomic species based on a combination of atomic mass, name, and type. This guess can be toggled on or off using the -guess option. If set to False and nonstandard atoms are present, MiMiCPy will exit with an error message. If one is not satisfied with the guessed elements, a file containing the list of all nonstandard atom types with the correct atomic elements can be specified with the -nsa option.

The PrepQM plugins for PyMOL and VMD have a very similar syntax and functionality as the command-line version. In the case of the VMD plugin:
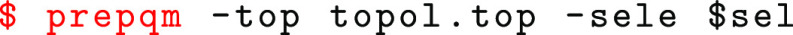
Here, the QM atoms are selected visually and/or using the VMD selection commands and entered into a named selection object called $sel. No coordinates need to be passed since they have already been loaded into VMD.

MiMiCPy can also be used as a Python library:
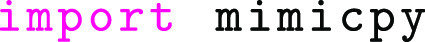
To load the topology and coordinate files into MiMiCPy, pass the file names to the DefaultSelector instance (or the VmdSelector or PyMOLSelector instances if desired):
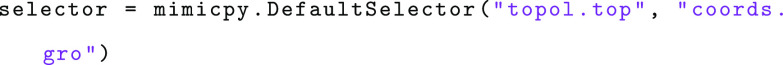
The selector instance is then used to instantiate the Preparation class to actually prepare the input files:
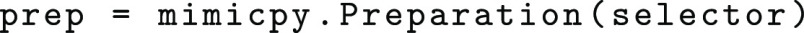


Atoms can be added and deleted to the QM region using the add() and delete() methods, respectively. Finally, the get_mimic_input() method can be called to generate the GROMACS index and the CPMD input instances:
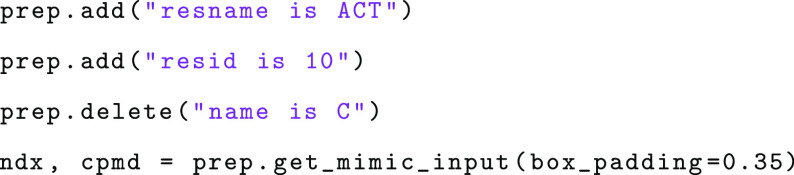
Parameters to change the way the CPMD file is written (e.g., box_padding to specify a minimal distance in nanometers between the QM atoms and the QM box boundaries) can be passed to get_mimic_input(). The resulting Script-type instances ndx and cpmd can be used to explore the properties in a “pythonic” way. For example, to print the total net charge of the QM region, reported as the CHARGE parameter in the &SYSTEM section of the CPMD input file, one can type:
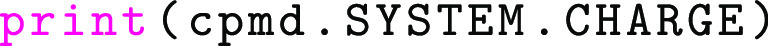


Further usage examples covering all MiMiCPy features in more detail can be found at https://mimic-project.org/.

## Applications

Here, we describe using the MiMiCPy command-line tools for a small molecule in water with the solute as the QM part and a protein in solution with covalent bonds that cross the QM-MM boundary.

### Case 1: Acetone in Water

Here, we setup a QM/MM simulation of an acetone molecule (QM subsystem) surrounded by water molecules (MM subsystem). The full system is equilibrated at the MM and QM/MM levels. Usually, the first step consists of an annealing simulation, where the temperature of the QM/MM system is smoothly decreased by removing the excess kinetic energy (released due to the relaxation from the MM to the optimal QM geometry) from the system. The input files for the annealing of this system are prepared using the MiMiCPy PrepQM tool. The following files have to be passed to PrepQM (see the Supporting Information for more details):

1. The GROMACS topology file topol.top and the MM equilibrated coordinate file coords.gro.
2. A “template” CPMD input file template.inp with only the &CPMD and &DFT sections filled to instruct CPMD to perform annealing.
3. A text file pp_info.dat, reporting pseudopotential details (like pseudopotential filenames, LMAX, LOC, etc.) for each element in the system.
4. A GROMACS simulation parameter file mimic.mdp with generic instructions to perform a MD run.

These four files are passed to MiMiCPy PrepQM with the command:
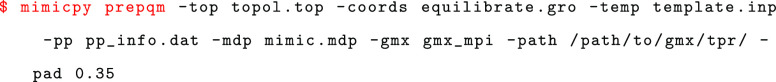


The acetone molecule is a nonstandard molecule; consequently, its atom definitions in the topology do not contain information about the species. The atomic elements are correctly guessed by PrepQM. The user is then asked to select, in an interactive environment, the atoms to be included in the QM region. The following commands can be entered to select the acetone molecule:
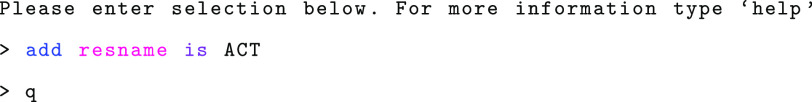


A new file cpmd.inp is created with the &ATOMS and the &MIMIC sections filled up from template.inp. The path to the GROMACS tpr file is also included when it is passed through the -path option of PrepQM. The pseudopotential information (specified with the -pp option) is included as specified in pp_info.dat. The QM system charge and size are calculated, and a value of 0.35 nm (as specified in the -pad option) is added to the QM box in all directions to comply with the requirement of the Poisson solver (Martyna and Tuckerman method) for isolated systems of CPMD. In practice, a larger value would have to be passed, depending on the system under consideration. The GROMACS index and tpr files are also generated.

### Case 2: The IDH1 Enzyme

The isocitrate dehydrogenase 1 enzyme (IDH1) from *Escherichia coli* in complex with the isocitrate ligand (ICT) and the cofactor NADP^+^ (PDB ID: 4AJ3)^27^ is first equilibrated at the MM level. Then, to run QM/MM, ICT and two residues (Arg 100 and Arg 109) involved in ligand binding are included in the QM region. This leaves NADP^+^ in the MM region, a nonstandard molecule with no atom species information in the topology file. This cannot be fixed by PrepQM, since it can only fix missing information for atoms in the QM region. CPMD does not have the species information on NADP^+^, possibly leading to segmentation fault errors. This can be avoided before launching PrepQM by running:
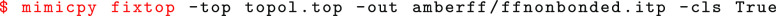


FixTop guesses missing atomic species information in the topology file (this includes atoms in the MM region, which PrepQM will not fix) and prints a consolidated [ atomtypes ] section into a GROMACS .itp file. The easiest way to incorporate this information into an existing GROMACS force field is to write it to the ffnonbonded.itp file containing the [ atomtypes ] definition of the whole system for all default GROMACS force fields. A copy of the AMBER force field directory is created locally under amberff/. FixTop replaces the [ atomtypes ] section in amberff/ffnonbonded.itp with the updated one containing all species information and clears other [ atomtypes ] sections from the topology (as -cls was specified). This can now be passed to PrepQM:
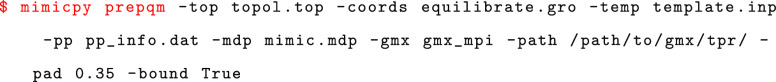
and typing in the interactive session:
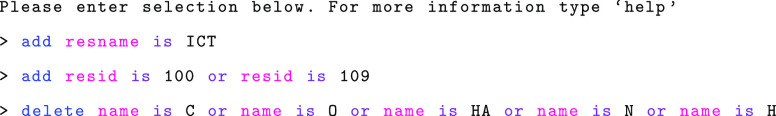
ICT and the (side chains of) two amino acid residues (with residue IDs 100 and 109) are included in the QM region without the backbone atoms.

The syntax used in the PrepQM command in this case is similar to the previous example, apart from an extra -bound option. The two amino acid residues included in the QM region are part of the protein, and hence, the QM-MM boundary cuts through covalent bonds with a QM and an MM atom on either side. These QM atoms need to be treated in a special way in order to saturate all the open valences in the QM region. One approach is to use the boundary-pseudoatom scheme where open-valence QM atoms are described through a special monovalent pseudopotential.^28^ When the -bound option is turned on, PrepQM will automatically detect the QM atoms with open valence and modify the CPMD input file accordingly.

The CPMD input file obtained in the output of the PrepQM subcommand can be edited further. We would like to constrain the distance between the atom H1 of ICT and the atom C4N of NADP^+^ to its current value. The following block has to be added to the &ATOMS section:
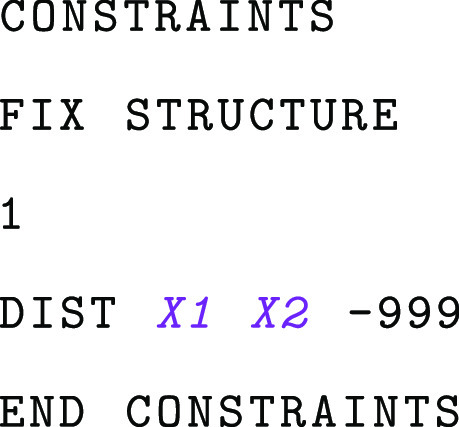
where in place of the placeholders 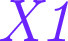 and 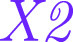, we should insert the CPMD indices of the two atoms involved in the constraint. Since ICT is in the QM region, we can look into the &MIMIC section of the CPMD input file to obtain the CPMD index of the H1 atom. However, as NADP^+^ is instead in the MM region, it is very difficult to obtain the CPMD index of the C4N atom by inspection of the input files alone. The MM atoms are grouped by species in a nonobvious way when transferring the data from GROMACS to CPMD. The CPMDid subcommand of MiMiCPy is provided to help in this context. It can be used to retrieve the CPMD indices corresponding to any atom in the topology file. For example, in our case, we launch the command:
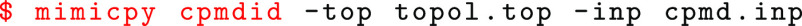
to enter in an interactive session where we provide the selection of the atoms we are interested in by using the usual selection language:
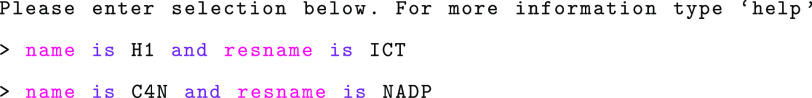
This will output the CPMD indices of the two atoms, which we can insert in the CONSTRAINTS block of the CPMD input. The indices can be printed in a table format (for debugging), list format (for quickly copying into the input), or as a range (for certain tasks like multiple thermostats). The printing format can be set with the -print option.

## Conclusions

We have presented MiMiCPy, a companion tool of MiMiC. The code simplifies the preparation and debugging of input files via a user-friendly interface. It provides an extensive list of command-line tools. PrepQM allows the generation of CPMD input files and GROMACS tpr files from the GROMACS topology and coordinate files. An easy-to-use selection language allows the selection and design of QM regions. The correct guess of atomic species from the MM topology is checked. Further tools to facilitate the interconvertibility between MM and QM engines are provided.

A plugin version of PrepQM for PyMOL and VMD allows the selection of visually complex QM regions. MiMiCPy can also be used as a Python library, allowing one to develop complex workflows to set up MiMiC-based QM/MM simulations. The package has been designed with a modular and object-oriented approach. This allows one (i) to easily support new topology and coordinate file formats from different programs, when they become available in MiMiC; (ii) to develop new tools as MiMiC expands its functionalities.

Applications to acetone and the IDH1 enzyme in water illustrate how users can expedite the setup, reducing human error by automating the procedure.

## Data Availability Statement

Releases of MiMiCPy are made available in the PyPI repository (https://pypi.org/project/mimicpy/). The source is available on GitLab at https://gitlab.com/MiMiC-projects/mimicpy, published under the GNU Lesser General Public License version 3 or later (LGPLv3+). Installation guides, tutorials, and other documentation are available at https://mimic-project.org/. Additional information about online documentation and software needed for running MiMiC-based QM/MM simulations is provided in the Supporting Information.

## Supporting Information Available

Further details on the topology and other input files mentioned in the Applications section, information about available online documentation, and a brief introduction to the MiMiC framework for QM/MM simulations (PDF)
