# Supplementary material for: MiMiCPy: An Efficient Toolkit for MiMiC-Based QM/MM Simulations
Source: J Chem Inf Model. 2023 Feb 22;63(5):1406–12. doi: 10.1021/acs.jcim.2c01620 (PMC10015468; doi:10.1021/acs.jcim.2c01620)
Supplement: Supplementary file 1 — ci2c01620_si_001.pdf [file ci2c01620_si_001.pdf]

Supporting Information: MiMiCPy: An Efficient Toolkit for MiMiC-Based QM/MM Simulations

Bharath Raghavan,[†,‡] Florian K. Schackert,[†,‡] Andrea Levy,[¶] Sophia K. Johnson,[¶] Emiliano Ippoliti,[†] Davide Mandelli,[*,†] Jógvan Magnus Haugaard Olsen,[§] Ursula Rothlisberger,[¶] and Paolo Carloni[*,†,‡]

†*Computational Biomedicine, Institute of Advanced Simulations IAS-5/Institute for Neuroscience and Medicine INM-9, Forschungszentrum Jülich GmbH, Jülich 52428, Germany*

‡*Department of Physics, RWTH Aachen University, Aachen 52074, Germany*

¶*Laboratory of Computational Chemistry and Biochemistry, Institute of Chemical Sciences and Engineering, École Polytechnique Fédérale de Lausanne (EPFL), CH-1015 Lausanne, Switzerland*

§*DTU Chemistry, Technical University of Denmark, DK-2800 Kongens Lyngby, Denmark*

E-mail: d.mandelli@fz-juelich.de; p.carloni@fz-juelich.de

Solvated Acetone Topology

We include here the files used to run Case 1 discussed in the Example Applications section.

The contents of `topol.top` are given below.

```
#include "amber03.ff/forcefield.itp"
#include "acetone.itp"
#include "amber03.ff/spc.itp"

[ system ]
    Generated by gmx solvate in water

[ molecules ]
    ACT          1
    SOL        506
```

The contents of `acetone.itp` are given below. This includes only the sections necessary to run the MiMiCPy command. Sections like `[ bonds ]`, `[ dihedrals ]`, etc. are not required by MiMiCPy PrepQM, only by GROMACS.

```
[ atomtypes ]
    c3          c3      0.0000  0.0000   A    3.39967e-01   4.57730e-01
    hc          hc      0.0000  0.0000   A    2.64953e-01   6.56888e-02
    c            c      0.0000  0.0000   A    3.39967e-01   3.59824e-01
    o            o      0.0000  0.0000   A    2.95992e-01   8.78640e-01

[ moleculetype ]
    ACT         3

[ atoms ]
    1            c       1     ACT      C1       1     0.81020   12.000000
    2           c3       1     ACT      C2       2    -0.47670   12.000000
```

```
3          c3      1    ACT     C3      3   -0.47670  12.000000
4           o      1    ACT     O1      4   -0.58640  16.000000
5          hc      1    ACT     H1      5    0.12160   1.000000
6          hc      1    ACT     H2      6    0.12160   1.000000
7          hc      1    ACT     H3      7    0.12160   1.000000
8          hc      1    ACT     H4      8    0.12160   1.000000
9          hc      1    ACT     H5      9    0.12160   1.000000
10         hc      1    ACT     H6     10    0.12160   1.000000
```

Template CPMD Input

A "template" CPMD input file can be passed to PrepQM. Here commands for a CPMD run (like the type of run, level of theory, etc.) can be given, and PrepQM will combine this with the QM atoms and other MiMiC information into a single CPMD input. This will allow fast generation of full CPMD input files for MiMiC runs. The contents of `template.inp` from cases 1 and 2 are given below. This includes all the CPMD commands not generated by PrepQM.

```
&MIMIC
    LONG-RANGE COUPLING
    FRAGMENT SORTING ATOM-WISE UPDATE
        100
    CUTOFF DISTANCE
        20.0
    MULTIPOLE ORDER
        3
&END

&CPMD
    MOLECULAR DYNAMICS CP
```

```
    NEW CONSTRAINTS
    ISOLATED MOLECULE
    QUENCH BO
    ANNEALING IONS
        0.99
    TEMPERATURE
        300
    EMASS
        600.0
    TIMESTEP
        5.0
    MAXSTEP
        10000
&END

&SYSTEM
    POISSON SOLVER TUCKERMAN
    SYMMETRY
        0
    CUTOFF
        70.
&END

&DFT
    FUNCTIONAL BLYP
&END
```

Pseudopotential Data File

The pp_info.dat file is a text file reporting pseudopotential details (like pseudopotential filenames, `LMAX`, `LOC`, etc.) for each element in the system. This will allow PrepQM to automat-

ically fill in these details for each atom in the CPMD input file. The data for each element is given in separate lines, and each line has the following format:

```
<element> <pp filename> <boundary pp filename> <labels> <lmax> <loc>
```

A "-" can be used to skip any field in this file. For the solvated acetone system, the contents of pp_info.dat are given below.

```
C C_MT_BLYP.psp C_GIA_DUM_AN_BLYP KLEINMAN-BYLANDER P -
H H_MT_BLYP.psp - KLEINMAN-BYLANDER S -
O O_MT_BLYP.psp - KLEINMAN-BYLANDER P -
```

IDH1 Topology Preparation

The IDH1 enzyme was discussed in Case 2 of the Example Applications section. The crystal structure was downloaded from the RCSB PDB database with the ID 4AJ3.[S1] The force field parameters for $NADP^+$ cofactor were obtained from a previous study in the AMBER topology format.[S2] The force field parameters for the isocitrate ligand were generated using the Generalised Amber Force Field,[S3] and partial charges were parameterized using the RESP method at HF/6-31G* level of theory. This was done using the ANTECHAMBER software package, which outputs the topology in the AMBER format.[S4]

The python package ACPYPE was used to convert the AMBER topology format of isocitrate and $NADP^+$ into the GROMACS format.[S5] The topology of the IDH1 protein was then prepared using the `gmx pdb2gmx` utility program, with parameters from the AMBER 99SBildn force field.[S6] This was combined with the ligand and cofactor parameters to obtain the full system topology.

Online Documentation

Documentation on the different ways of using the PrepQM command and on the usage of the other command line tools (CPMD2Coords, FixTop, CPMDid and Geom2Coords) is

available on the MiMiC website mimic-project.org. Specifically, the "MiMiCPy handbook" section contains a detailed guide on the usage of the VMD/PyMOL plugins, the MiMiCPy python library, and the command line tools for a solvated acetone.

The MiMiC Framework

The MiMiC framework consists of two libraries: (i) the main MiMiC library, which contains optimized routines for fast computation of the interactions between different *subsystems*, and (ii) the MiMiC communication library, which is a light-weight library that is used to exchange information between the MiMiC library and *external programs*. We refer to Ref.[S7] for more detailed information. MiMiC-based QM/MM simulations currently use modified versions of GROMACS 2021[S8] and CPMD 4.3,[S9] serving as the two external programs dealing with the MM and QM subsystems, respectively. All the software is open source and freely available. The official website mimic-project.org can be consulted for detailed step-by-step instructions guiding through the installation process and for user support.

At the time of writing, installing the software needed to run MiMiC-based QM/MM simulations involves the following four main steps:

1. Download and install the MiMiC communication library

2. Download and install the MiMiC library

3. Download GROMACS and the corresponding MiMiC patch, apply the patch and install

4. Download CPMD and the corresponding MiMiC patch, apply the patch and install

Steps 1 and 2 must be performed strictly in this order. After that, GROMACS and CPMD can be patched and compiled. To facilitate the installation process, the MiMiC interface is expected to be part of future releases of the two programs. We are also planning to add EasyBuild and Spack packages to facilitate the installation of the MiMiC framework on supercomputers.

So far the MiMiC framework has been (locally) installed and tested on the following HPC supercomputers: the JUWELS, JURECA, and JUSUF supercomputers hosted at the Jülich Supercomputing Center (Forschungszentrum Jülich, Germany), the CLAIX supercomputers hosted at RWTH Aachen (Aachen, Germany), the SuperMUC-NG supercomputer hosted at the Leibniz Supercomputing Center (Münich, Germany), the Galileo supercomputer hosted at CINECA (Casalecchio di Reno, Italy), the PIZ DAINT supercomputer hosted at CSCS, the Swiss National Supercomputing Centre (Lugano, Switzerland), and the LUMI supercomputer hosted by the LUMI consortium at CSC's data center (Kajaani, Finland).

References

(S1) Gonçalves, S.; Miller, S. P.; Carrondo, M. A.; Dean, A. M.; Matias, P. M. Induced fit and the catalytic mechanism of isocitrate dehydrogenase. *Biochemistry* **2012**, *51*, 7098–7115.

(S2) Holmberg, N.; Ryde, U.; Bülow, L. Redesign of the coenzyme specificity in l-Lactate dehydrogenase from Bacillus stearothermophilus using site-directed mutagenesis and media engineering. *Protein Engineering, Design and Selection* **1999**, *12*, 851–856.

(S3) Wang, J.; Wolf, R. M.; Caldwell, J. W.; Kollman, P. A.; Case, D. A. Development and testing of a general amber force field. *Journal of Computational Chemistry* **2004**, *25*, 1157–1174.

(S4) Wang, J.; Wang, W.; Kollman, P. A.; Case, D. A. Automatic atom type and bond type perception in molecular mechanical calculations. *Journal of Molecular Graphics and Modelling* **2006**, *25*, 247–260.

(S5) Sousa Da Silva, A. W.; Vranken, W. F. ACPYPE - AnteChamber PYthon Parser interfacE. *BMC Research Notes* **2012**, *5*, 1–8.

(S6) Hornak, V.; Abel, R.; Okur, A.; Strockbine, B.; Roitberg, A.; Simmerling, C. Comparison of multiple Amber force fields and development of improved protein backbone parameters. *Proteins* **2006**, *65*, 712–725.

(S7) Olsen, J. M. H.; Bolnykh, V.; Meloni, S.; Ippoliti, E.; Bircher, M. P.; Carloni, P.; Rothlisberger, U. MiMiC: A Novel Framework for Multiscale Modeling in Computational Chemistry. *Journal of Chemical Theory and Computation* **2019**, *15*, 3810–3823.

(S8) Abraham, M. J.; Murtola, T.; Schulz, R.; Páll, S.; Smith, J. C.; Hess, B.; Lindah, E. GROMACS: High performance molecular simulations through multi-level parallelism from laptops to supercomputers. *SoftwareX* **2015**, *1-2*, 19–25.

(S9) Hutter, J.; Alavi, A.; Deutsch, T.; Bernasconi, M.; Goedecker, S.; Marx, D.; Tuckerman, M.; Parrinello, M. CPMD, IBM Corp 1990-2022, Copyright MPI für Festkörperforschung Stuttgart 1997-2001. `http://www.cpmd.org/`.
